# Protective defunctioning stoma in bowel segmental resection at the time of total hysterectomy for endometriosis: when less is more

**DOI:** 10.1007/s00404-024-07629-5

**Published:** 2024-07-12

**Authors:** Carolina Dolci, Yohan Kerbage, Alessandro Ferdinando Ruffolo, Massimo Candiani, Anne Gandon, Chrystèle Rubod

**Affiliations:** 1 Centre Hospitalier Universitaire de Lille, Service de Chirurgie Gynécologique, 59000 Lille, France; 2 Gynecology/Obstetrics Unit, IRCCS San Raffaele Scientific Institute, Via Olgettina 60, 20132 Milan, Italy; 3 Faculté de Médecine, Université de Lille, 59000 Lille, France

**Keywords:** Bowel endometriosis, Total hysterectomy, Segmental resection, Post-operative complications, Preventing stoma

## Abstract

**Purpose:**

To compare postoperative complications in women undergoing total hysterectomy with segmental resection (TH-SR) for intestinal endometriosis with or without protective defunctioning stoma (PDS) confection.

**Methods:**

Retrospective cohort study conducted at the Gynecologic department of University Hospital of Lille (France) from January 2008 to January 2022 in patients undergone TH-SR for bowel endometriosis.

**Results:**

100 women were considered for the analysis. PDS were performed in 56 women. The rate of rectal resections was significantly higher in the PDS group (*p* = *0.03*). The mean operative time, AAGL scores and length of hospital stay were significantly higher in the PDS group (*p* = *0.002*). The rate of grade III complication according to Clavien-Dindo classification was higher in the PDS group (*p* = *0.03*). Among digestive complications, one case of anastomosis leakage (1.8%) and one case of recto-vaginal fistula (2.3%) was recorded in the non-PDS group, 4 cases of anastomosis stenosis were recorded in the PDS group (7.1%). Persisting bladder atony requiring self-catheterization over one month was the most common disturb (4.6% in the non-PDS group and 7.1% in the PDS group, *p* = *0.58*). The distance of digestive lesion from anal margin was the only risk factor for digestive complications, persistent bladder atony, Clavien-Dindo IIIA and IIIB complications at the multivariate analysis (*p* = *0.04* and *p* = *0.06* respectively).

**Conclusion:**

No statistically significant differences were found in the rate of digestive complications in case of total hysterectomy and concomitant segmental resection when performing or not preventing stoma.

## What does this study add to the clinical work

**Table:** 

Avoid the realization of PDS at the time of radical surgery for intestinal endometriosis, especially in case of endometriotic lesions not affecting the lower rectum.	

## Introduction

Bowel endometriosis affects 5–12% of patients with endometriosis, with 90% of the lesions seen in the recto-sigmoid region [1–3]. Symptoms like dysmenorrhea, dyschezia, dyspareunia, constipation, tenesmus are commonly associated with bowel endometriosis, often determining a detrimental impact on patients’ quality of life [4, 5].

In patients with any symptoms of intestinal obstruction, or in those which medical therapy was ineffective, surgical treatment is considered. The three suggested surgical approaches for bowel endometriosis include the shaving technique, disc excision and segmental resection(SR). In the absence of clear agreement among surgeons, the choice of the technique depends on the site, dimension, number and percentage of lumen stenosis of the lesions [6, 7]. When a SR is performed, a protective defunctioning stoma(PDS) could be considered, even if its effectiveness in endometriosis context remains debated[8]. The increase in surgical complexity, the need for a second surgery and the considerable discomfort for the patients should be also considered [9–11]. The new French guidelines for the management of colorectal endometriosis consider PDS only in case of low rectal lesion and concomitant colpectomy [12].

Alternatively, an omental flap interposition(epiploplasty) between the digestive scar and the vaginal suture was proposed, even if it is not always feasible and its efficacy in this context was not proven [13].

The aim of the present study, therefore, was to evaluate the role of PDS on the occurrence of postoperative complications in patients requiring total hysterectomy and concomitant SR(TH-SR) for intestinal endometriosis.

## Materials and methods

This retrospective study was carried out between January 2008 and January 2022 at the Gynecologic department of University Hospital of Lille, France. We included symptomatic women affected by histologically confirmed bowel endometriosis, aged more than 18 years, with no more pregnancy desire, undergoing TH-SR. Patients submitted to subtotal hysterectomy, with malignancy at the histologic specimen, with preexistent PDS or lost at follow-up were excluded. All the included women signed a written informed consent to record their data for scientific purposes. The study was conducted in accordance with the Declaration of Helsinki and approved by the Ethical Review Committee of the University Hospital of Lille (CEROG 2022-GYN-1203).

Data regarding patients age, medical and obstetric history, previous abdominal surgery, previous medical and surgical treatment for endometriosis, symptoms related to endometriosis were collected from medical records.

The initial diagnosis of bowel endometriosis was made through a pelvic magnetic resonance imaging (MRI). Intestinal endometriosis was defined as deep endometriosis with infiltration of at least the muscolaris [14]. A rectal endoscopic sonography or computed tomography (TC) were then performed to better identify the involvement of bowel wall layers, the distance from anal marge and the percentage of lumen stenosis. All radiological examinations were performed or at least reviewed in our institution. All the surgical indications were validated after discussion in an endometriosis multidisciplinary setting.

During surgery, the abdominal and pelvic cavities were explored to identify all endometriotic lesions. Hysterectomy with bilateral salpingectomy with or without ovariectomy was completed. The decision to perform ovariectomy was based on patients age and ovarian findings. Bowel SR were performed in an interdisciplinary approach together with colorectal surgeons [8, 15]. PDS with or without epiploplasty was performed to protect the anastomosis, according to intra-operative findings. All the others DIE lesions were excised, to accomplish the objective of complete surgery. Urinary tract endometriotic lesions were treated according to their anatomic extent [16].

Data including duration of surgery, blood loss, size of histological specimens, length of hospital stay, surgical complications, reoperations and hospital readmissions were retrieved from medical reports. The surgical complexity was estimated according to the American Association of Gynecologic Laparoscopists (AAGL) score from surgical reports [17].

Patients were systematically reviewed both by gynecologic and gastro-intestinal surgeon at 6 weeks after surgery. In case of normal follow-up, women were addressed to general gynecologists at 3 months. In case of unfavorable clinical evolution, closer follow-up with the surgeons were planned. In the case of PDS, the closure was performed after a contrast enema to exclude subclinical leaks.

Based on the surgical decision to perform or not the PDS, we retrospectively divided our study population in two group: the PDS group and the non-PDS group.

Surgical complications were classified according to the Clavien Dindo (CD) classification [18]. De-novo voiding dysfunction requiring self-catheterization lasting more than 1 month was considered as a major complication.

The primary endpoint of this study was to compare the rate of surgical complication when PDS was performed or not. The secondary endpoint was the assessment of the potential risk factors in the occurrence of surgical complications.

### Statistical analysis

Statistical Package for Social Science (SPSS) version 21.0 was used to perform data analysis. The Kolmogorov–Smirnov test was used to analyze the normal distribution of the variables (*p*-value > 0.05). Continuous variables were expressed as mean and standard deviation (SD) or as median and interquartile range(IQR). For continuous variables the Student’s t test (for normally distributed data) and Mann–Whitney U test (for non‐normally distributed data) for independent samples were adopted as appropriate. For categorical variables, the statistical significance of differences in distribution was tested with Pearson χ2 test or with the exact Fisher test as appropriate. The statistical analysis was conducted at a 95%confidence level and a *p*‐value < 0.05 was considered statistically significant. Logistic regression was applied to test the association between risk factors for intestinal complications and bladder atony. Exploratory univariate analyses were initially applied to all variables, and variables that had a significant association with the adopted scores at univariate analysis (95% confidence level) were eventually included in the multivariate analyses (95% confidence level).

## Results

**Fig. 1:**
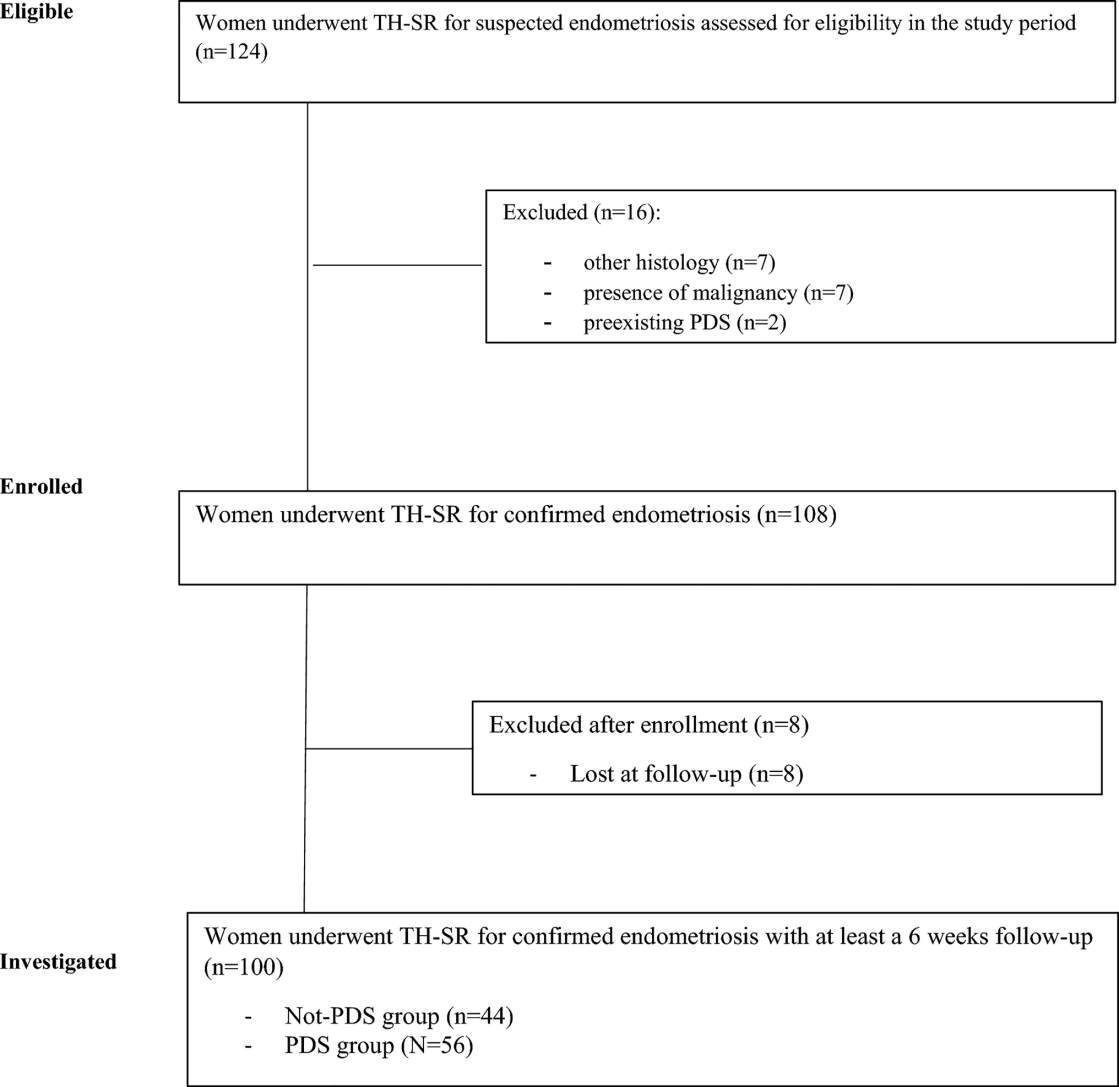
Strengthening the reporting of observational studies in epidemiology (STROBE) flow chart of study design. *TH-RS: total hysterectomy and concomitant segmental resection, PDS protective defunctiongn stoma

During the study period, 124 women underwent TH-SR for suspected endometriosis. Of these, seven women were excluded because the suspicion of endometriosis was not confirmed at the histology, seven women were excluded because an ovarian malignancy was found at the histological specimen, two women were excluded because of preexisting PDS and eight women were lost at follow-up. The remaining 100 women were considered for the analysis. A PDS were performed in 56 women(Fig. 1).

The baseline characteristics of the included women are reported in Table 1. The majority of lesions were seen in the sigmoid (70.4% in the non-PDS group and 58.9% in the PDS group, *p* = *0.13*) and recto-sigmoid region (29.5% in the non-PDS group and 53.6.9% in the PDS group, *p* = *0.01*). The lesions were mostly unifocal and with a median size major of 3 cm at the MRI.

**Table 1:** Characteristic of the study population at baseline

	Non ileostomy (*n* = 44)	Ileostomy (*n* = 56)	*p* value
Age, years, mean, ± SD	41.4 ± 5.1	40.4 ± 5.0	0.30
Parous women, *n* (%)	33 (75)	33 (58.9)	0.09
Parity, median (IQR)	2 (1–2)	2 (1–2)	0.51
Cesarean section, *n* (%)	16 (36.4)	9 (16.1)	0.02
Previous gynecological surgery, *n* (%)			
1 surgery	31 (70.4)	43 (76.8)	0.47
≥ 2 surgeries	17 (38.6)	23 (41.1)	0.80
Previous endometriosis surgeries, *n* (%)	24 (54.5)	32 (57.1)	0.79
1 Surgery	16 (36.4)	16 (28.6)	0.40
≥ 2 surgeries	8 (18.2)	16 (28.6)	0.22
≥ 1 laparotomy	6 (13.6)	14 (25)	0.15
Symptoms, *n* (%)			
Dysmenorrhea	23 (52.3)	36 (64.3)	0.22
Dyspareunia	18 (40.9)	24 (42.8)	0.84
Chronic pelvic pain	30 (68.2)	33 (58.9)	0.34
Dysuria	12 (27.3)	11 (19.6)	0.36
Dyschezia	15 (34.1)	16 (28.6)	0.55
Alternance constipation/diarrhea	23 (52.3)	23 (41,1)	0.26
Occlusion/subocclusion	4 (9.1)	3 (5.3)	0.69
Abnormal vaginal bleeding	18 (40.9)	14 (25)	0.09
MRI number of digestive lesions, *n* (%)			
No lesion	2 (4.5)	0 (0)	0.19
1 lesion	33 (75)	36 (64.2)	0.25
2 lesions	9 (20.4)	19 (33.9)	0.13
3 lesions	0 (0)	1 (1.8)	0.99
MRI localization of digestive lesions, *n* (%)			
Rectum	5 (11.4)	11 (19.6)	0.26
Rectum-sigma	13 (29.5)	30 (53.6)	0.01
Sigma	31 (70.4)	33 (58.9)	0.13
Colon	1 (2.3)	0 (0)	0.44
Cecum	1 (2.3)	1 (1.8)	0.99
Small bowel	0 (0)	1 (1.8)	0.99
MRI size of digestive lesions, mm, median (IQR)	35 (25.25–47.75)	32 (23–50)	0.80
MRI distance of digestive lesion from anal margin, cm, mean ± SD	15.52 ± 4.1	13.77 ± 4.9	0.12
ColoTDM digestive lesion with stenosis ≥ 50%, *n* (%)	8 (18.2)	21 (37.5)	0.03

*SD*: standard deviation;* IQR*: interquartile range;* MRI*: magnetic resonance imaging;* TDM*: tomodensiometry

The intra and peri-operative data are shown in Table 2. The surgical approach was more common laparoscopic (79.5% and 73.2% respectively, *p* = *0.46*), with 3 (5.3%, *p* = *0.25*) laparo-conversion in the PDS group. In line with the MRI findings, the bowel lesions were mainly unifocal (75% in the non-PDS group and 58.9% in the PDS group) and seen more commonly in the sigmoid (75% in the non-PDS group and 71.4% in the PDS group*, p* = *0.68*) and in the rectum (40.9% in the non-PDS group and 57.1% in the PDS group*, p* = *0.10*), but a higher proportion of cecal and small-bowel lesions were found at the time of surgery (2.3% and 11.4% respectively in the non-PDS group and 7.1% and 10.7% in the PDS group). The bowel segmental resections were more common single (90.1% in the non-PDS group and 76.8% in the PDS group, *p* = *0.10*), recto-sigmoid (84% in the non-PDS group and 85.7% in the PDS group, *p* = *0.82*), with a mean total length of 13.7 ± 7.9 cm and 16.9 ± 11.5 cm respectively (*p* = *0.11*). The rate of rectal resections was significantly higher in the PDS-group (*p* = *0.03*). Ureterolysis and DIE nodules excision were the most common additional procedures performed at the time of surgery (93% and 54.5% respectively in the non-PDS group, 92.8% and 46.4% in the PDS group, *p* = *0.94* and *p* = *0.42*). Ureteral reimplantation were only performed in the PDS group (*p* = *0.008*). There was not significant difference in the performing of the epiploplasty between the two groups (*p* = *0.79*), whereas the placement of abdominal drainage was significantly more common in the PDS group (*p* = *0.001*). The mean operative time, the mean AAGL scores and the mean length of hospital stay were significantly higher in the PDS group (*p* = *0.002*).

**Table 2:** Intra- and peri-operative characteristics of the study population

	Non ileostomy (*n* = 44)	Ileostomy (*n* = 56)	*p* value
Laparoscopy, *n* (%)	35 (79.5)	41 (73.2)	0.46
Laparotomy, *n* (%)	7 (15.9)	12 (21.4)	0.59
Robotic, *n* (%)	2 (4.6)	3 (5.3)	0.85
Laparotomic conversion, *n* (%)	0 (0)	3 (5.3)	0.25
Number of intraoperative bowel lesions, *n* (%)			
1 lesion	33 (75)	33 (58.9)	0.21
2 lesions	9 (20.4)	17 (30.4)	
3 lesions	2 (4.6)	6 (10.7)	
Localization of intraoperative bowel lesions, *n* (%)	18 (40.9)	32 (57.1)	0.10
Rectum	33 (75)	40 (71.4)	0.68
Sigma	1 (2.3)	4 (7.1)	0.38
Cecum	0 (0)	1 (1.8)	0.99
Appendix	5 (11.4)	6 (10.7)	0.91
Small bowel			
Number of bowel resection, *n* (%)			0.10
Single resection	40 (90.1)	43 (76.8)	
Double resection	4 (9.9)	13 (23.2)	
Localization of bowel resection, *n* (%)			
Rectum	0 (0)	6 (10.7)	**0.03**
Rectum-sigma	37 (84)	48 (85.7)	0.82
Sigma	6 (13.6)	3 (5.3)	0.17
Ileo-cecal	3 (6.8)	10 (17.8)	0.13
Small bowel	2 (4.5)	2 (3.6)	0.99
Intestinal resection total length, cm, mean ± SD	13.7 ± 7.9	16.9 ± 11.5	0.11
Other surgeries, *n* (%)			
Ureterolysis	41 (93)	52 (92.8)	0.94
Ureteral reimplantation	0 (0)	8 (14.3)	**0.008**
Partial cystectomy	0 (0)	3 (5.3)	0.25
Exeresis of DIE nodule	24 (54.5)	26 (46.4)	0.42
Rectal shaving	10 (22.7)	5 (8.9)	0.06
Rectal discoid resection	0 (0)	1 (1.8)	0.99
Appendicectomy	4 (9.1)	3 (5.3)	0.69
Colpectomy	3 (6.8)	9 (16.1)	0.21
Epiploplasty, *n* (%)	24 (54.5)	32 (57.1)	0.79
Abdominal drainage, *n* (%)	17 (38.6)	39 (69.6)	**0.001**
Operative time, min, mean ± SD	327.3 ± 74.9	392.7 ± 85.7	**0.002**
AAGL score, mean ± SD	39.3 ± 10.42	47.6 ± 14.0	**0.002**
AAGL stage, *n* (%)			0.58
Stage III	2 (4.6)	1 (1.8)	
Stage IV	42 (95.4)	55 (98.2)	
Length of hospital stay, days, mean ± SD	6.2 ± 3.0	7.8 ± 3.6	**0.02**

*SD*: standard deviation; American Association of Gynecological Laparoscopists

No significant difference was found between the two groups in the rate of grade I and grade II complications, whereas the rate of grade III complication according to Clavien-Dindo classification was higher in the PDS group (*p* = *0.03*), as reported in Table 3. No grade IV and V complications were reported. Among the digestive complications, we recorded one case of anastomosis leakage in the non PDS group (2.3%), one case of recto-vaginal fistula in the non-PDS group (1.8%) and 4 cases of anastomosis stenosis in the PDS group (7.1%). The anastomosis leakage required reoperation under general anesthesia with peritoneal toilette and the confection of a defunctioning stoma. The anastomosis stenosis needed dilatation through a rectosigmoidscopy under general anesthesia. The recto-vaginal fistula required a laparotomic reoperation under general anesthesia with colo-anal stoma in two steps according to Baulieux technique [19] and a new vaginal suture.

**Table 3:** Clavien-Dindo classification of post-operative complications. Number of women presenting complications (*n*, %), and raw number of complications (*n*, %)

Grade	Non ileostomy (*n* = 44)	Ileostomy (*n* = 56)	Treatment	*p* value
Grade I, *n* (%)	5 (11.4)	10 (17.8)		0.12
Incomplete cicatrisation of the anastomosis	0 (0)	1 (1.8)	Delayed closure of ileostomy	
Compartmental syndrome	1 (2.3)		Hydration	
Rhabdomyolysis	0 (0)		Hydration	
Functional ileum	0 (0)		Conservative treatment	
Deficit brachial plexus	0 (0)		Spontaneous regression	
Neural symptom s lower limbs	0 (0)		Physiotherapy	
Haematuria	0 (0)		Bladder lavages	
Fever ndd	1 (2.3)		Antipyretics	
Vault vaginal ulcer	1 (2.3)		Conservative treatment	
Vaginal granuloma	1 (2.3)		Conservative treatment	
Hypokalaemia	1 (2.3)		Electrolytes	
Abdominal wall hematoma	0 (0)	1 (1.8)	Conservative treatment	
Grade II, *n* (%)	13 (29.5)	22 (39.3)		0.31
Functional ileum	2 (4.6)	8 (14.3)	Nasogastric tube	
Acute pyelonephritis	2 (4.6)	2 (3.6)	Antibiotics	
Inferior urinary tract infection	4 (9.1)	7 (12.5)	Antibiotics	
Bladder injury	0 (0)	1 (1.8)	Indwelling catheter	
Bladder atony	5 (11.4)	7 (12.5)	Indwelling/intermittent catheter	
Adrenal insufficiency	1 (2.3)	0 (0)	Cortisone	
Postoperative anaemia	1 (2.3)	4 (7.1)	Martial therapy/Transfusion	
Deep venous thrombosis	0 (0)	1 (1.8)	Anticoagulant	
Vena porta thrombosis	0 (0)	1 (1.8)	Anticoagulant	
Abdominal wall infection	1 (2.3)	7 (12.5)	Antibiotics	
Grade III, *n* (%)	3 (6.8)	13 (23.2)		**0.03**
Grade III A	0 (0)	1 (1.8)	Nasogas	0.99
Atelectasis	0 (0)	1 (1.8)	Fibroscopic pulmonary expansion	
Grade III B	3 (6.8)	12 (16.1)		0.05
Ureteral fistula	1 (2.3)	0 (0)	Ureteral resection, vesico-ureteral reimplantation and double J	
Ureteral injury	0 (0)	1 (1.8)	Ureteral resection, vesico-ureteral reimplantation and double J	
Umbilical hernia and sub-occlusion	1 (2.3)	0 (0)	Hernioplasty	
Recto-vaginal fistula	0 (0)	1 (1.8)	Surgical reparation	
Anastomosis leakage	1 (2.3)	0 (0)	Surgical reparation	
Anastomosis stenosis	0 (0)	4 (7.1)	Dilatation or bowel resection with anastomosis	
Ileostomy herniation	0 (0)	4 (7.1)	Hernioplasty at time of ileostomy closure	
Hematoma of the ileostomy site	0 (0)	1 (1.8)	Drainage	
Abdominal wall abscess	0 (0)	1 (1.8)	Drainage	
Presacral abscess	0 (0)	1 (1.8)	Drainage	
Compartmental syndrome	0 (0)	1 (1.8))	Fasciotomy	
Pneumothorax	0 (0)	1 (1.8)	Fibroscopic Pulmonary drainage	

The median timing of ileostomy closure in the PDS group was 8 week [IQR 7–10] and the median time of follow-up was 4 (3–8.25) and 6 (4–14) months in the non PDS group and PDS group respectively (*p* = *0.06*).

Persisting bladder atony requiring self-catheterization over one month after surgery was the most common disturb (4.6% in the non-PDS group and 7.1% in the PDS-group, *p* = *0.58*).

The exploratory univariate analyses evaluating risk factors for intestinal complications (Table 4) found not significant associations except for the MRI distance of digestive lesion from anal margin (*p* = *0.04*) and the length of bowel resection (*p* = *0.06*), but at the multivariate analysis, only the MRI distance of digestive lesion from anal margin was significative (*p* = *0.04*). The exploratory univariate analyses and multivariate analysis evaluating risk factors for persistent bladder atony, Clavien-Dindo IIIA and IIIB complications (Table 5) found not significant associations except for the MRI distance of digestive lesion from anal margin (*p* = *0.06*).

**Table 4:** Risk factors for intestinal complications (recto-vaginal fistula, anastomosis leakage anastomosis stenosis)

	Univariate		Multivariate	
	HR	*p*-value	HR	*p*-value
Age, years	0.9 (0.8–1.1)	0.44		
Previous gynecological surgery ≥ 2	0.2 (0.1–1.4)	0.18		
Previous endometriosis surgery	0.5 (0.1–1.9)	0.44		
ColoTDM digestive lesion with stenosis ≥ 50%	3.1 (0.8–11.5)	0.15		
MRI distance of digestive lesion from anal margin ≤ 10 cm	5.6 (1.4–21.8)	**0.04**	5.9 (1.1–32.4)	**0.03**
Laparotomic approach	0.6 (0.1–3.5)	0.61		
Colpectomy	1.3 (0.2–8.9)	0.77		
Rectal shaving and discoid resection	1.2 (0.2–7.9)	0.84		
≥ 2 Intraoperative intestinal localization	0.8 (0.2–3.2)	0.75		
≥ 2 Intestinal resection	0.8 (0.1–5.2)	0.84		
Total resection length, cm	1.0 (1.0–1.1)	**0.06**	1.0 (0.9–1.1)	0.06
Ileostomy	5.2 (0.8–31.5)	0.13		
Epiploplasty	0.6 (0.1–2.1)	0.47		
Drainage	1.0 (0.2–3.9)	0.94		

*MRI*: magnetic resonance imaging; *TDM*: tomodensiometry

**Table 5:** Risk factors for persistent bladder atony and Clavien-Dindo IIIA and IIIB grade

	Univariate		Multivariate	
	HR	*p*-value	HR	*p*-value
Age, years	0.9 (0.9–1.0)	0.19		
Previous gynecological surgery ≥ 2	0.5 (0.2–1.3)	0.23		
Previous endometriosis surgery	1.3 (0.6–2.9)	0.61		
ColoTDM digestive lesion with stenosis ≥ 50%	1.4 (0.6–3.2)	0.50		
MRI distance of digestive lesion from anal margin ≤ 10 cm	3.5 (1.3–9.7)	**0.04**	3.5 (1.1–11.7)	**0.04**
Laparotomic approach	1.6 (0.6–3.9)	0.41		
Ureterolysis	1.9 (0.3–11.8)	0.54		
DIE excision	1.8 (0.8–4.2)	0.22		
Colpectomy	0.8 (0.2–3.1)	0.80		
Rectal shaving and discoid resection	0.7 (0.2–2.8)	0.69		
≥ 2 Intraoperative intestinal localization	0.9 (0.4–2.3)	0.94		
≥ 2 Intestinal resection	0.4 (0.1–1.7)	0.31		
Total resection length, cm	1.0 (0.9–1.1)	0.11		

*MRI*: magnetic resonance imaging; *TDM*: tomodensiometry

## Discussion

Our results suggested that, in case of TH-SR for endometriosis, performing PDS does not reduce the risk of severe peri-operative (grade IIIA and IIIB according to CD classification) complications, especially digestive complications.

These results are more remarkable if we consider the context of concomitant colpectomy, reported to be one of the main risk factors for digestive complications after intestinal resection, together with low rectal lesions (less than 5 cm from the anal verge) [20].

The rate of grade III complications was higher in PDS group. This difference can be explained by the more complicated surgical interventions in this group, demonstrated by the longer operative time, the needs of ureteral reimplantations, the significantly higher number of rectal lesions (*p* = 0.03), the higher AAGL scores, the longer hospital stay, and by the presence of stoma itself. Indeed, specific stoma-related complications include high output and electrolyte disturbance, skin complications and parastomal hernias. Indeed, concerns about the medium/longer term risks associated with PDS, including functional digestive complications due to the microbiota intestinal alterations and long-term sequelae of high output (e.g., chronic kidney disease), are rising also in the field of rectal cancer surgery [21].

Very few data exist in literature on the benefices of performing PDS in the case of SR at the time of TH for endometriosis. Laskmann in 2006 [22] and Pikron et al. in 2009 described the feasibility of laparoscopic colorectal resection at the time of TH for endometriosis in a single patient respectively [23]. In a retrospective study on 29 symptomatic women affected by colorectal endometriosis, Darai et al. confirmed the feasibility of laparoscopic TH-SR, performing only 3 PDS. One case of anastomosis leakage was registered, but it was not specified if the patients was submitted to PDS or not [24]. Lim et al., in 2011 carried out a retrospective study on 18 women submitted to low anterior resection with sigmoid rectal anastomosis and TH for endometriosis comparing the robotic-assisted approach to the laparotomic approach. The authors did not perform PDS and registered two cases of recto-vaginal fistula only in the laparotomic group, demonstrating the feasibility of robotic approach [25]. In 2019, Boudy et al. performed a retrospective study on 27 patients undergone laparoscopic TH-SR for endometriosis, performing a prevescical peritoneum interposition between vagina and digestive scars, with only a case of recto-vaginal fistula registered [13]. In 2022, Roman et al. retrospectively compared postoperative complications and rectovaginal fistula rate in women undergoing excision of rectovaginal endometriosis requiring concomitant excision of rectum and vagina during two time periods with differing policies for preventive stoma confection. No significant differences were found concerning risk of rectovaginal fistula (9.2% and 11.1%, *p* = *0.80*) during the first and the second period [26].

Therefore, to our knowledge, this study represents the largest cohort in literature on this topic and the first study aiming to evaluate the role of performing PDS in terms of surgical complications when a radical surgery for intestinal endometriosis was realized. Demonstrating the absence of significant differences in the rate of digestive complications when performing PDS, it is possible to exclude its presumed protective role and avoid its realization in case of radical surgery for intestinal endometriosis, at least for lesions above the rectum.

Additionally, all the included patients received a complete preoperative imaging work-up by experienced radiologists, the decision to operate all our patients was discussed in a multidisciplinary fashion and all the surgical interventions were performed in an endometriosis referral center by expert gynecologic surgeons in collaboration with the colorectal surgery team. Other strengths of our study are the strict inclusion and exclusion criteria, the long duration of follow-up and low rate of patients lost at follow-up.

The main limitations of our study are the retrospective design with all its intrinsic issues and the monocentric setting. The long study period over than 10 years is another important limitation of the study, because potentially responsible for difference in patients management over time, but it was necessary to reach our sample size. In addition, in the absence of randomization, the decision to perform PDS was not random. Based on the population’s surgical characteristics, PDS was significantly more performed in case of rectal resection and, even if not significantly, in case of colpectomy. Therefore, the interest of the PDS as a preventive measure in the event of a rectal resection cannot be assessed due to the systematic use of a stoma in these cases.

## Conclusions

In the absence of clear guidelines in the management of patients undergone TH-SR, our results suggest the absence of significant differences in the rate of digestive complications when performing or not PDS, even if the utility of PDS in low rectal lesion was impossible to estimate. Thus, it is possible to exclude the presumed protective role of PDS and avoid its realization at the time of radical surgery for intestinal endometriosis, especially in case of lesions not affecting the lower rectum. Larger, randomized studies reporting post-operative complications after performing TH-SR with or without PDS are required.

## Data Availability

The datasets analyzed during the current study are not publicly available due to privacy concerns but are available from the corresponding author on reasonable request.
